# On the Management of Hyperglycaemia in Critically Ill Patients Undergoing Surgery

**DOI:** 10.4021/jocmr604w

**Published:** 2012-07-20

**Authors:** Iakovos Nomikos, Maria Kyriazi, Dimitra Vamvakopoulou, Andreas Sidiropoulos, Athanasios Apostolou, Aspasia Kyritsaka, Evangelos Athanassiou, Nikolaos C. Vamvakopoulos

**Affiliations:** a Departments of Surgery (B’ Unit), “METAXA” Cancer Memorial Hospital, Piraeus, Greece; b Departments of Surgery, University of Thessalia Medical School, Biopolis 41110, Larisa, Greece; c Biology and Genetics, University of Thessalia Medical School, Biopolis 41110, Larisa, Greece; d Cytogenetics and Medical Genetics Laboratory, University Hospital, 41110 Thessalia, Greece

**Keywords:** Hyperglycaemia, Surgical complications, Critically ill patients

## Abstract

Hyperglycaemia is a major health risk and a negative determinant of surgical outcome. Despite its increasing prevalence, the limited treatments for restoration of normoglycaemia make its effective management a highly complex individualized clinical art. In this context, we review the mechanisms leading to hyperglycaemic damage as the basis for effective management of surgical complications of diabetic and non diabetic critically ill patients.

## Introduction

Hyperglycaemia resulting from stress-induced impairment of glucose tolerance is common in critically ill patients, even those without diabetes mellitus. The hyperglycaemic condition appears to aggravate the untoward effects of concomitant illness, leading to prolonged hospital stay, higher health care resource utilization and cost [1] and is associated with increased in-hospital morbidity and mortality for a variety of medical conditions and surgical procedures [2, 3]. The clinician is faced with the dual challenge to maintain normoglycaemia during surgery and minimize the untoward effects of hyperglycaemia [4]. Further development of appropriate marker-guided targeted interventions for selection of effective combination of insulin administration modes to meet individual patient needs may offer promising treatment options. We review the clinical biology of hyperglycaemia and present blood glucose control management options of this metabolic condition for the protection of critically ill patients undergoing surgery.

## Evidence of Hyperglycaemic Damage

The development of most adverse outcomes of hyperglycaemia, including increased inflammatory mediators, endothelial cell dysfunction, defects in immune function, haemostatic deregulations leading to thromboses and increased oxidative stress from elevated reactive oxygen species (ROS) and superoxide anion production, has been widely studied [5, 6]. Furthermore numerous clinical parameters are also being adversely affected by hyperglycaemia in critically ill patients like duration of stay in the intensive care unit (ICU), duration of ventilatory support and inotropic and vasopressor treatment, number of transfusions, duration of antibiotic therapy, presence of critical illness polyneuropathy, mean APACHE II score and cumulative TISS score and survival [7]. In the cardiovascular system, hyperglycaemia contributes to myocyte apoptosis, impaired ischemic preconditioning and increased infarct size [8]. Tight control of blood glucose appears to alleviate hyperglycaemic damage and restore islet-graft function by ensuring normal development of new vessels and preservation of endothelial lining in experimental animals [5, 9, 10].

Prevention of hyperglycaemia-induced dysfunction in cellular systems that allow insulin-independent glucose uptake, like central and peripheral nervous system, endothelial, epithelial and immune cells, would explain some of the protective effects of insulin therapy in critically ill patients [11]. In clinical practice, achievement of normoglycaemia before a surgical procedure is carried out, ensures proper activation of the basic steps of the healing process like normal vascularization and inflammatory response [12, 13].

While some experts use routine administration of intensive insulin therapy to normalize glucose levels of hyperglycaemic patients in the ICU, others raise serious concerns over the actual definition of the optimal glucose level, the accuracy of measurements, the resources required to attain tight glycaemic control and the impact of tight glycaemic control across the heterogeneous ICU population including patients with diabetes, previously undiagnosed diabetes or stress-induced hyperglycaemia [14-16].

## Mechanism of Hyperglycaemic Damage

Mainly hexosamine, advanced glycation end product (AGE) formation and diacylglycerol protein kinase C pathways, activated by increased availability of the glycolytic metabolites glyceraldehydes-3-phosphate and fructose-6-phosphate, are implicated in the pathogenesis of hyperglycaemia induced vascular damage. The lipid-soluble thiamine derivative benfotiamine inhibits the enzyme transketolase, which converts glyceraldehydes-3-phosphate and fructose-6-phosphate into pentose-5-phosphates and other sugars as well as hyperglycaemia-associated NF-kB activation, and might be clinically useful in preventing development and progression of diabetic complications [17].

In critical illness the generation of neuroendocrine responses by acute insults like the stress of surgery and anaesthesia, trauma or sepsis, alters carbohydrate metabolism, causes excessive secretion of counter-regulatory hormones like catecholamines, cortisol, glucagon, and growth hormone, promotes insulin resistance, increases hepatic glucose production via enhanced glycogenolysis and gluconeogenesis and impairs peripheral glucose utilization and insulin action. Four glucose transporters, GLUT-1, GLUT-2, GLUT-3 and GLUT-4, facilitate insulin-independent glucose transport in these tissues. In critical illness, cytokines, VEGF, TGF-β and hypoxia appear to up regulate the expression and membrane localization of GLUT-1 and -3 transporters in different cells types. This “stress response” may overrule the normal protection of cells against hyperglycaemia, thus allowing cellular glucose overload. Hence, all organ systems that take up glucose independent of insulin may be at high risk for direct glucose toxicity by the consistently attained high levels of these regulators during critical illness [18].

Insulin binding to its receptors activates two main branching pathways: a) the mitogenic or “growth signal” and b) the metabolic. The former affects cell proliferation and the latter activates protein kinase B (PKB) leading to nitric oxide (NO) generation and apoptosis via metabolic mobilization of glucose, lipid, and protein stores. In patients with long standing diabetes, the metabolic consequences of insulin resistance are mediated predominantly by abnormalities along the metabolic pathway of insulin signalling caused primarily by exogenous insulin administration. However, a disrupted metabolic pathway does not mean that the mitogenic insulin signalling path will also be equally unresponsive. Compensatory hyperinsulinemia may thus still exert mitogenic actions in certain cell types while the metabolic actions of insulin are suppressed. This discrepancy may occur in vascular smooth muscle cells and in specific capillary endothelial cells of patients with type-2 diabetes and obesity. Whether insulin resistance is similarly “selective” during critical illness and hyperinsulinemia exerts adverse effects through this pathway in the acute setting of severe illness is presently unknown [18].

## Clinical Prevention of Hyperglycaemic Damage

Clinical evidence suggests that glycaemic control is independent predictor of patient survival and normoglycaemia is beneficial on surgical outcome [19-21]. Achievement of a normoglycaemic environment long before a surgical procedure is carried out ensures proper activation of the basic steps of the healing process like normal vascularization and inflammatory response [6, 13]. One study reported that only 40% of 25 ICU patients achieved target blood glucose levels using accepted insulin infusion, initiation and maintenance algorithms [22], while another including 50 patients reported considerably broader success rates ranging from 25% to over 70% [23]. Recent multi-center trials of intensive glycaemic control in critically ill patients using more (4.4 - 6.1 mmol/L) [24] or less (4-8 mmol/L) [25] stringent glucose targets yielded similar patient mortality rates [25-30].

Recent guidelines for the glycaemic control of ICU patients with severe sepsis recommend the use of validated protocols for insulin dose adjustment to maintain blood glucose < 150 mg/dL (8.3mmol/L) [31, 32] Implementation of analogous insulin infusion algorithms to secure tight insulin control in a broader spectrum of critically ill patients in the ICU however, led to increased rates of hypoglycaemia of unclear clinical significance [33]. The increased variability of circulating glucose during critical illness has negative effect on therapeutic intervention and deleterious impact on survival, particularly of non-diabetic hyperglycaemic patients. Van den Berghe reported the incidence of hypoglycaemia (< 40 mg/dL or 2.2 mmol) associated with tight glycaemic control in a medical ICU, as high as 18.7%. Application of various approaches and computer-based algorithms may definitely improve this high incidence. The impact of hypoglycaemia, particularly in patients with septic shock and those with neurological compromise, warrants further evaluation [34-36].

The clinical benefits of intensive insulin therapy for prevention of hyperglycaemia after surgery are yet controversial. One study reported reduced morbidity and death of critically ill patients undergoing primary cardiac surgery [23] while another concluded that intensive insulin therapy during cardiac surgery does not reduce perioperative morbidity or death [27]. More recently, comprehensive clinical implementation of a strict insulin infusion protocol for glycaemic control of cardiac surgery patients also proved ineffective when individual risk factors of patient hyperglycaemia could not be met [37].

The significantly higher asymmetric dimethylalanine (ADMA) plasma levels recorded in ICU patients who died compared with survivors, coupled to the potent modulation of this marker by intensive insulin therapy, made ADMA monitoring valuable predictor of adverse ICU outcome [7]. Compared to monitoring advanced glycation end products (AGES) [18], plasma ADMA levels are better selectors of accepted insulin initiation and maintenance infusion algorithms for tight control of blood glucose in critically ill patients undergoing surgery with minimal complications [38-40]. A recent clinical study using insulin-free, metformin-based regimens to achieve durable glycaemic control, defined by glycated haemoglobin level of less than 8%, in young patients with type 2 diabetes, confirmed the expected from assisted reproduction gender-related effectiveness of the treatment and highlighted the complex nature of primary patient response leading to loss of glycaemic control or sustained metabolic decompensation requiring insulin administration [41].

The primal and highly complex nature of glucose homeostasis may explain the controversial and occasionally confusing results of clinical studies aiming to prevent or minimize hyperglycaemia and its complications [42]. The complex regulation of gene expression of key genetic components of the stress response system is in line and supports this hypothesis [43-47]. Thus, a recent in vitro study showed that the increase of glucose concentrations exerted multiple opposing effects on cellular and immunologic parameters, like substantial impairment of the functional capacity of the innate immune response, reflected by formation of potent killing ROS, along with paradoxical attenuation of pro-inflammatory cytokine release and enhancement of phagocytosis. The opposing findings at the cellular level might contribute to the clinical controversies that have thrown recent practice and protocols into disarray. Expert physician judgment and experience are essential in the imminent treatment of one of medicine’s most important recent clinical debates, as additional cellular mechanistic studies and clinical trials continue to emerge [48].

On the other hand, the results of a multi-center, randomized, controlled trial comparing sensor-augmented pump therapy with a regimen of multiple daily insulin injections in adult and pediatric patients with inadequately controlled type 1 diabetes showed that continuous glucose monitoring can be an effective tool for the intensification of glucose control in patients with type 1 diabetes without incurring an increased risk of hypoglycaemia [49].

Clearly, glycaemic control of critically ill patients is a highly individualized clinical art that requires coordinated management by health care professionals. Future improvements in the management of these patients are likely to profit from advances in: 1. Targeted prospective clinical research protocols aiming to delineate the systemic effects of hyperglycaemia and insulin therapy and 2. Predictive marker-guided insulin administration protocols able to achieve and maintain glycaemic targets with minimal side effects like hypoglycaemia.

## References

[1] Estrada CA, Young JA, Nifong LW, Chitwood WR, Jr (2003). Outcomes and perioperative hyperglycemia in patients with or without diabetes mellitus undergoing coronary artery bypass grafting. Ann Thorac Surg.

[2] Krinsley JS (2003). Association between hyperglycemia and increased hospital mortality in a heterogeneous population of critically ill patients. Mayo Clin Proc.

[3] Noordzij PG, Boersma E, Schreiner F, Kertai MD, Feringa HH, Dunkelgrun M, Bax JJ (2007). Increased preoperative glucose levels are associated with perioperative mortality in patients undergoing noncardiac, nonvascular surgery. Eur J Endocrinol.

[4] Jesson ME (2003). Glucose control during cardiac surgery: how sweet it is. J Thorac Cardiovascul Surg.

[5] Nomikos I, Kalogerakos K, Athanasiou E, Plakokefalos E, Sioutopoulou D, Satra M, Vamvakopoulos NC (2008). Role of hyperglycemia in isogeneic islet transplantation: an experimental animal study. Exp Clin Endocrinol Diabetes.

[6] Nomikos IN, Malizos C, Vamvakopoulos NC (2006). Protective and damaging aspects of healing: A review. Wounds.

[7] Siroen MP, van Leeuwen PA, Nijveldt RJ, Teerlink T, Wouters PJ, Van den Berghe G (2005). Modulation of asymmetric dimethylarginine in critically ill patients receiving intensive insulin treatment: a possible explanation of reduced morbidity and mortality?. Crit Care Med.

[8] Das UN (2001). Is insulin an antiinflammatory molecule?. Nutrition.

[9] Biarnes M, Montolio M, Nacher V, Raurell M, Soler J, Montanya E (2002). Beta-cell death and mass in syngeneically transplanted islets exposed to short- and long-term hyperglycemia. Diabetes.

[10] Merino JF, Nacher V, Raurell M, Biarnes M, Soler J, Montanya E (2000). Optimal insulin treatment in syngeneic islet transplantation. Cell Transplant.

[11] Furnary AP, Zerr KJ, Grunkemeier GL, Starr A (1999). Continuous intravenous insulin infusion reduces the incidence of deep sternal wound infection in diabetic patients after cardiac surgical procedures. Ann Thorac Surg.

[12] Clayton SB, Mazur JE, Condren S, Hermayer KL, Strange C (2006). Evaluation of an intensive insulin protocol for septic patients in a medical intensive care unit. Crit Care Med.

[13] Nomikos IN, Vamvakopoulos NC (2001). Correlating functional staging to effective treatment of acute surgical illness. Am J Surg.

[14] Biolo G, De Cicco M, Lorenzon S, Dal Mas V, Fantin D, Paroni R, Barazzoni R (2008). Treating hyperglycemia improves skeletal muscle protein metabolism in cancer patients after major surgery. Crit Care Med.

[15] Ali NA, O'Brien JM, Jr, Dungan K, Phillips G, Marsh CB, Lemeshow S, Connors AF, Jr (2008). Glucose variability and mortality in patients with sepsis. Crit Care Med.

[16] Egi M, Bellomo R, Stachowski E, French CJ, Hart GK, Hegarty C, Bailey M (2008). Blood glucose concentration and outcome of critical illness: the impact of diabetes. Crit Care Med.

[17] Hammes HP, Du X, Edelstein D, Taguchi T, Matsumura T, Ju Q, Lin J (2003). Benfotiamine blocks three major pathways of hyperglycemic damage and prevents experimental diabetic retinopathy. Nat Med.

[18] Nomikos IN, Sidiropoulos A, Vamvakopoulou DN, Athanassiou E, Mouzas OD, Perrakis N, Tsilimingas N (2009). Surgical complications of hyperglycaemia. Curr Diabetes Rev.

[19] Heuer JG, Sharma GR, Zhang T, Ding C, Bailey DL, Stephens EJ, Holmes KC (2006). Effects of hyperglycemia and insulin therapy on outcome in a hyperglycemic septic model of critical illness. J Trauma.

[20] Hagiwara S, Iwasaka H, Hasegawa A, Koga H, Noguchi T (2008). Effects of hyperglycemia and insulin therapy on high mobility group box 1 in endotoxin-induced acute lung injury in a rat model. Crit Care Med.

[21] Yung M, Wilkins B, Norton L, Slater A (2008). Glucose control, organ failure, and mortality in pediatric intensive care. Pediatr Crit Care Med.

[22] McMullin J, Brozek J, McDonald E, Clarke F, Jaeschke R, Heels-Ansdell D, Leppert R (2007). Lowering of glucose in critical care: a randomized pilot trial. J Crit Care.

[23] Shulman R, Finney SJ, O'Sullivan C, Glynne PA, Greene R (2007). Tight glycaemic control: a prospective observational study of a computerised decision-supported intensive insulin therapy protocol. Crit Care.

[24] van den Berghe G, Wouters P, Weekers F, Verwaest C, Bruyninckx F, Schetz M, Vlasselaers D (2001). Intensive insulin therapy in critically ill patients. N Engl J Med.

[25] Finney SJ, Zekveld C, Elia A, Evans TW (2003). Glucose control and mortality in critically ill patients. JAMA.

[26] Subramaniam B, Panzica PJ, Novack V, Mahmood F, Matyal R, Mitchell JD, Sundar E (2009). Continuous perioperative insulin infusion decreases major cardiovascular events in patients undergoing vascular surgery: a prospective, randomized trial. Anesthesiology.

[27] Gandhi GY, Nuttall GA, Abel MD, Mullany CJ, Schaff HV, O'Brien PC, Johnson MG (2007). Intensive intraoperative insulin therapy versus conventional glucose management during cardiac surgery: a randomized trial. Ann Intern Med.

[28] Marfella R, Portoghese M, Ferraraccio F, Siniscalchi M, Babieri M, Di Filippo C, D'Amico M (2009). Thiazolidinediones may contribute to the intramyocardial lipid accumulation in diabetic myocardium: effects on cardiac function. Heart.

[29] Finfer S, Chittock DR, Su SY, Blair D, Foster D, Dhingra V, Bellomo R (2009). Intensive versus conventional glucose control in critically ill patients. N Engl J Med.

[30] Van den Berghe G, Wouters PJ, Bouillon R, Weekers F, Verwaest C, Schetz M, Vlasselaers D (2003). Outcome benefit of intensive insulin therapy in the critically ill: Insulin dose versus glycemic control. Crit Care Med.

[31] Krinsley JS (2008). Glycemic variability: a strong independent predictor of mortality in critically ill patients. Crit Care Med.

[32] Dellinger RP, Levy MM, Carlet JM, Bion J, Parker MM, Jaeschke R, Reinhart K (2008). Surviving Sepsis Campaign: international guidelines for management of severe sepsis and septic shock: 2008. Crit Care Med.

[33] Latham R, Lancaster AD, Covington JF, Pirolo JS, Thomas CS, Jr (2001). The association of diabetes and glucose control with surgical-site infections among cardiothoracic surgery patients. Infect Control Hosp Epidemiol.

[34] Vriesendorp TM, DeVries JH, Hoekstra JB (2008). Hypoglycemia and strict glycemic control in critically ill patients. Curr Opin Crit Care.

[35] Vriesendorp TM, DeVries JH, van Santen S, Moeniralam HS, de Jonge E, Roos YB, Schultz MJ (2006). Evaluation of short-term consequences of hypoglycemia in an intensive care unit. Crit Care Med.

[36] Brunkhorst FM, Engel C, Bloos F, Meier-Hellmann A, Ragaller M, Weiler N, Moerer O (2008). Intensive insulin therapy and pentastarch resuscitation in severe sepsis. N Engl J Med.

[37] Murphy MA, Whitman I, Campfield A, Moxey E, Haddad M, Whitman G (2010). Intense implementation of a strict insulin infusion protocol does not guarantee postoperative glycemic control. J Am Coll Surg.

[38] (2008). Standards of medical care in diabetes. Diabetes Care.

[39] Curfman GD, Morrissey S, Drazen JM (2008). Why doctors should worry about preemption. N Engl J Med.

[40] Fahy BG, Sheehy AM, Coursin DB (2009). Glucose control in the intensive care unit. Crit Care Med.

[41] (2012). A Clinical Trial to Maintain Glycemic Control in Youth with Type 2 Diabetes. N Engl J Med.

[42] Manolakis AC, Kapsoritakis AN, Tiaka EK, Sidiropoulos A, Gerovassili A, Satra M, Vamvakopoulou D (2011). TLR4 gene polymorphisms: evidence for protection against type 2 diabetes but not for diabetes-associated ischaemic heart disease. Eur J Endocrinol.

[43] Vamvakopoulos NC, Mayol V, Margioris AN, Chrousos GP (1992). Lack of dexamethasone modulation of mRNAs involved in the glucocorticoid signal transduction pathway in two cell systems. Steroids.

[44] Vamvakopoulos NV (1995). Sexual dimorphism of stress response and immune/ inflammatory reaction: the corticotropin releasing hormone perspective. Mediators Inflamm.

[45] Vamvakopoulos NC, Rojas K, Overhauser J, Durkin AS, Nierman WC, Chrousos GP (1993). Mapping the human melanocortin 2 receptor (adrenocorticotropic hormone receptor; ACTHR) gene (MC2R) to the small arm of chromosome 18 (18p11.21-pter). Genomics.

[46] Vamvakopoulos NC, Sioutopoulou TO (1994). Human corticotropin-releasing hormone receptor gene (CRHR) is located on the long arm of chromosome 17 (17q12-qter). Chromosome Res.

[47] Vamvakopoulos NC, Sioutopoulou TO, Durkin SA, Nierman WC, Wasmuth JJ, McPherson JD (1995). Mapping the human corticotropin releasing hormone binding protein gene (CRHBP) to the long arm of chromosome 5 (5q11.2-q13.3). Genomics.

[48] Qadan M, Weller EB, Gardner SA, Maldonado C, Fry DE, Polk HC, Jr (2010). Glucose and surgical sepsis: a study of underlying immunologic mechanisms. J Am Coll Surg.

[49] Bergenstal RM, Tamborlane WV, Ahmann A, Buse JB, Dailey G, Davis SN, Joyce C (2010). Effectiveness of sensor-augmented insulin-pump therapy in type 1 diabetes. N Engl J Med.

